# Intussusception Secondary to a Meckel's Diverticulum in an Adolescent

**DOI:** 10.1155/2011/623863

**Published:** 2011-09-25

**Authors:** John Morrison, Rebecca Jeanmonod

**Affiliations:** St. Luke's Hospital and Health Network 801 Ostrum Sreet Bethlehem, PA 18015, USA

## Abstract

A 13-year-old girl presented to the Emergency Department with vomiting and abdominal pain. On examination, she had only mild abdominal tenderness, but a mass was palpable in her right lower quadrant. Intussusception was diagnosed on ultrasound and confirmed on computed tomography (CT) scan, and operative findings revealed a jejunojejunal intussusception secondary to Meckel's diverticulum. Intussusception is a surgical abdominal emergency, which can present in all ages but is the most common reason for small bowel obstruction in childhood. It is a well-known cause of abdominal pain, vomiting, and bloody diarrhea in infancy but often not considered when evaluating the older child with similar symptoms. However, consideration of this diagnosis is important, as more than 1/3 of cases present beyond the age of 7. In older children, intussusception is more likely to be related to underlying pathology, such as Meckel's diverticulum, malignancy, or polyp. Intussusception should be on the differential in any patient with isolated abdominal complaints, and when it is diagnosed in an older child, it should be recognized that it is likely secondary to underlying pathology.

## 1. Introduction

Intussusception is a gastrointestinal condition describing the involution of a proximal portion of the bowel into a more distal portion, leading to inflammation and often bowel obstruction. It is a relatively common etiology of abdominal pain in children, with greatest incidence between 3 months and 6 years of age. Traditional teaching describes a patient within this age range with the triad of abdominal pain, vomiting, and bloody mucoid stools. However, these age ranges and classic symptoms represent only a subset of patients presenting with this condition. Since a missed diagnosis of intussusception may lead to bowel necrosis, perforation, sepsis, and death, it is critical for the emergency clinician to contemplate this diagnosis in children and adolescents who do not present with the typical signs and symptoms. We present the case of a patient beyond the conventional age range and without the classic symptom triad who was ultimately diagnosed with intussusception.

## 2. The Case

A 13-year-old female presented to the emergency department with the chief complaint of sudden onset abdominal pain beginning 10 hours prior to arrival. She stated the pain was located in her mid-abdomen and was pressure-like in nature, 6/10 in intensity, and constant with no radiation. It was not relieved with acetaminophen. The patient also reported nausea with multiple episodes of nonbloody emesis throughout the day. She denied fever, chills, diarrhea, constipation, dysuria, hematuria, or menstrual abnormalities, and her review of systems was otherwise negative. She denied any recent travel and reported no known sick contacts. The patient and her father reported no past medical problems nor relevant family history. 

On examination, the patient's blood pressure was 108/58 mmHg, her heart rate was 86 BPM, her respiratory rate was 16, her temperature was 96.8°F, and her oxygen saturation was 100% on room air. She was pale but non-toxic in appearance. Her head, neck and chest exams were within normal limits. The patient's abdominal exam was remarkable for mild suprapubic and left lower quadrant tenderness with no guarding or rebound. A deep firm mass was palpable above the pelvic brim to the right of midline. The patient had no McBurney's point tenderness. She was retching on occasion throughout exam. Laboratory data was remarkable for a white blood cell count of 15,000 96% neutrophils. Blood chemistries and urinalysis were both normal. 

**Figure 1:**
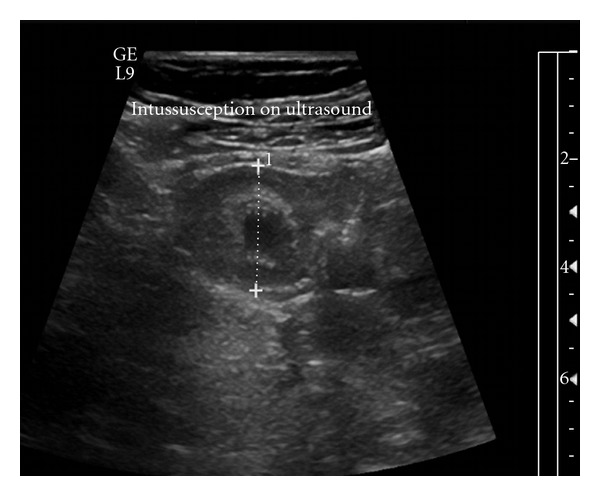
Transabdominal ultrasound with mass in right lower quadrant.

**Figure 2:**
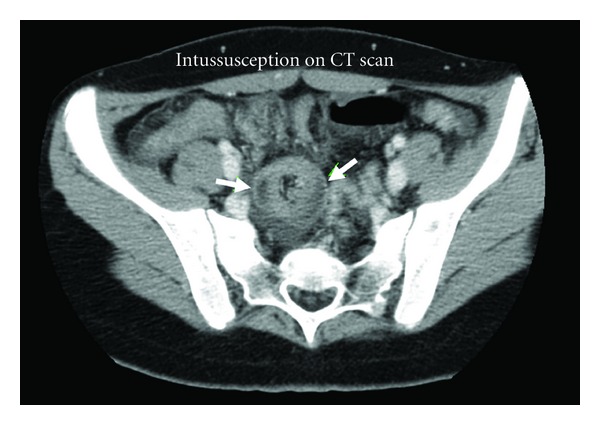
CT scan of abdomen showing intussuscepted bowel.

Because the mass was concerning for ovarian pathology, a transabdominal pelvic ultrasound was ordered to rule out possible ovarian torsion. Results revealed no evidence of ovarian torsion but showed a prominent bowel loop in the right lower quadrant anteromedial to the right ovary (Figure 1). At this time, the surgical team was consulted and the patient underwent a computed tomography (CT) scan of the abdomen and pelvis. This revealed a likely ileoileal intussusception with moderate dilation of the proximal small bowel and a moderate amount of pelvic free fluid (Figure 2). The patient underwent exploratory laparotomy and was found to have a mid-jejunal intussusception with a Meckel's diverticulum as the lead point. The diverticulum was resected, and the patient had an uncomplicated postoperative course.

## 3. Discussion

Intussusception is a condition in which one portion of the bowel, usually proximal to the ileocecal valve, invaginates into an adjacent segment. This process leads to bowel wall edema which progressively causes obstruction of venous outflow. The bowel becomes secondarily ischemic, which can eventually lead to necrosis and perforation. Ileocolic intussusceptions are the most common with ileoileal, cecocolic, colocolic, and, as in our patient, jejunojejunal, occurring less often [1].

The greatest incidence for intussusception is between 3 months and 6 years of age with a higher prevalence in males than females; however, it is not uncommon in older children [2]. A recent large retrospective study found that older children (aged 7–18 years) comprised up to 13% of all intussusceptions and adults made up 23% of the total. This further merits the need to consider this differential in all age groups [3]. Some large studies report that up to 10% of intussusceptions are secondary to a pathologic lead point, with increased risk between ages five and fourteen [4, 5]. After this age, the greater proportion of intussusceptions is due to underlying pathology, with only about 10% of cases considered idiopathic [6]. Specific lead points identified in the past include carcinoid tumors and leiomyoma, but intussusception can also be associated with generalized pathologies such as Peutz-Jeghers syndrome, Henoch Schonlein purpura, neutropenic colitis, cystic fibrosis, celiac disease, ascaris infection, and other malignancies [1, 4, 5, 7–9]. It may also occur after certain operative procedures [1, 8, 9]. There has been an association reported with the Rotavirus vaccine although additional research is needed [2, 10]. 

Although there are many lesions that give rise to intussusception, Meckel's diverticulum is the most common [4]. A Meckel's diverticulum is a remnant of the omphalomesenteric duct, which is normally obliterated by the 5th to 8th week of gestation. It is present in up to 22% of the population, but only 4% of those will have a complication in their lifetime [11, 12]. Those complications, when they occur, are most often bowel obstruction, intussusception, diverticulitis, and bleeding, with most obstructions secondary to intussusceptions [12, 13]. Clearly, since most intussusceptions are idiopathic in origin, and most Meckel's diverticuli are asymptomatic, the diagnosis of intussusception secondary to Meckel's diverticulum is very rare in all populations.

In spite of the rarity of this specific disease in this age group as presented in this case (one prior report by Pollack and Pender), it does serve to illustrate an important concept [8]. Traditional teaching and textbooks report intussusception as being a disease of children aged 2 months to 2 years most commonly presenting with the classic triad of vomiting, abdominal pain, and bloody stools. Unfortunately, these symptoms occur together in less than 25% of patients with intussusceptions [9, 10]. Furthermore, the “currant jelly” stool (a mixture of dark blood and stool) which is considered classic for intussusception is less likely to occur in older children as compared to infants and less likely to occur in those with small bowel intussusceptions [3, 6, 8]. Additionally, the ability to palpate a related abdominal mass or determine focal tenderness in a small child depends highly on the skill of the clinician and relaxation of the abdominal wall musculature [1]. This makes diagnosis difficult even when the provider is suspicious for the disease.

That said, it is common for physicians to fail to consider this diagnosis in patients with gastrointestinal symptoms outside the early childhood period even in the presence of known underlying pathology. Indeed, given that gastroenteritis is much more common in children and adults of all ages, it is easy to become complacent and assign that diagnosis to adolescents with atypical symptoms such as our patient, who had mild pain and vomiting but no diarrhea [10]. Other delays in diagnosis may be due to failing to recognize painless intussusceptions, dismissing of symptoms in older aged patients, failure to appreciate the typically atypical presentation of those with small bowel origins of intussusception, and failing to recognize the significance of bilious vomiting [14]. In addition, intussusception can occur postoperatively and symptoms are often dismissed as secondary to the initial intra-abdominal issue or as pain secondary to the procedure [9]. 

Although mortality and surgery rates have dropped over the past 50 years partly due to increased use of nonoperative reduction techniques and improved ability to diagnose intussusception with CT scans and ultrasound, all of these presuppose consideration of the diagnosis [14]. If intussusception is missed, it can be disastrous, progressing to bowel necrosis, perforation, sepsis, and death. Therefore, this is an important diagnosis to consider in all children with gastrointestinal complaints, particularly with symptoms that do not fit a classic gastroenteritis pattern.

## 4. Conclusion

Although there is no way to determine if intussusception is idiopathic versus secondary to a pathological lead point based on signs or symptoms, this delineation is irrelevant to the emergency department practitioner [9]. However, it is important for a clinician to contemplate this diagnosis not only in the neonate and infant but also in the less typical adolescent and adult patient. The patient described in this case, with only mild abdominal pain and isolated vomiting, illustrates that a broad differential should be entertained when addressing patients with gastrointestinal complaints and abdominal pain.
